# Correlations among the maternal healthcare services in Bangladesh: An application of joint modelling technique

**DOI:** 10.1016/j.heliyon.2023.e22453

**Published:** 2023-11-24

**Authors:** Kakoli Rani Bhowmik, Sumonkanti Das, Unnati Rani Saha, Ruhul Amin, Md Atiqul Islam

**Affiliations:** a Department of Bio-Medical Data Sciences, Leiden University Medical Centre, Leiden, the Netherlands; b School of Demography, Australian National University, Canberra, Australia; c Department of Public Health, Erasmus MC, University Medical Center Rotterdam, the Netherlands; d Bangladesh Institute of Governance and Management (BIGM), Dhaka, 1207, Bangladesh; e Department of Statistics, Jagannath University, Dhaka, Bangladesh

**Keywords:** Antenatal care, Delivery in private facilities, Caesarean section, Inter-correlation, Joint model

## Abstract

**Background:**

Caesarean section (C-section) in Bangladesh have received great attention as the number has been amplified during the last two decades. The question arises whether this rise has a correlation with other maternal healthcare services and/or has been influenced by their predictors.

**Objective:**

The main objectives of this study are to assess correlations among the maternal healthcare indicators—antenatal care use, childbirth in private facilities, and childbirth through C-section—and identify their associated predictors in Bangladesh through the development of an appropriate cluster-adjusted joint model that accounts for inter-correlation among the indicators in the same cluster.

**Design:**

The 2019 Bangladesh Multiple Indicator Cluster Survey data have been utilized in this study. Separate generalized linear mixed models developed for the three outcome variables are combined into a joint model by letting cluster-specific random effects be in association.

**Findings:**

The joint model shows that the number of antenatal cares is fairly positively correlated with delivery in private facilities and C-section, while the latter two are strongly positively correlated. Household socio-economic condition, women and their partners' education, women's exposure to mass media, place of residence, religion, and regional settings have significant influence on the joint likelihood of receiving antenatal care, choosing a private health facility for birth, and opting for C-section birth.

**Key conclusions and implications:**

The rising rate of C-section delivery over time is alarming for Bangladesh to achieve the World Health Organization target of 10–15 %. The joint model reveals that the rising rate of C-sections may be correlated with the choice of a private health facility as the delivery place. The study findings also suggest that maternal childbirth care is private-dominant and predominantly utilized by urban women with better education and higher socio-economic status. The policy should focus on strengthening the public health sector while also keeping importance in increasing coverage of maternal care services among the less well-off.

## 1 Introduction

The Caesarean section (hereafter defined as C-section) has witnessed a rising trend over the past decades globally [1,2]. In 1985, the World Health Organization (WHO) stated that the C-section rates for any country should not be any higher than 10–15 % of all births [3,4]. In spite of that, developed and developing countries are hardly seen to follow this WHO recommended threshold [5,6]. Bangladesh is one of those countries with the current C-section rate of 31 % of the total births [[7], [8], [9]], which is an abnormal rise of about ten-fold from the rate of 3 % in 2000 [1,10,11] and an alarming rate for the country. Though the underlying risk factors for the increased C-section were deeply investigated and revealed in past studies [12,13], less is known about the correlated variables of the C-section around the pregnancy, which might have an influence on the continuation of the rising C-section rate in the country.

Several studies conducted in Bangladesh have investigated the association between maternal healthcare services antenatal care (hereafter defined as ANC), delivery in private facilities (hereafter defined as DPF), and the increased C-section [1,[14], [15], [16], [17], [18], [19]] utilizing the data either from the Bangladesh Demographic and Health Survey (BDHS) or from the Bangladesh Multiple Indicator Cluster Survey (MICS) and have concluded that the likelihood of C-section among the pregnant women increases with the number of ANC visits and the utilization of private facilities (hereafter defined as PF) for their childbirth. Moreover, the number of ANC visits among the expectants increases the likelihood of undergoing institutional delivery, including DPF [15,16,18,19]. In those studies, maternal healthcare indicators ANC, DPF, and C-section were treated as a single outcome variable and analysed utilizing the simplest univariate approach, ignoring their interdependency and inter-correlation in the datasets. However, these outcome variables are correlated due to the clustered or hierarchical nature of the BDHS and MICS datasets and this correlation may play a significant role in the increase of C-section rate in the country. As the inter-correlations among these variables were not controlled in the previous studies, this can also lead to bias in the prediction of the associated predictors, which in turn may result in bias in the policy recommendations [[20], [21], [22], [23]].

To the best of our knowledge, there is still no study in the literature that focuses on the correlation among these three outcomes and assesses the effect of shared predictors of the correlated outcomes on the increase in C-section rate. Therefore, a systematic joint study on ANC, DPF, and C-section is indeed essential to account for the interdependency and intercorrelation among the outcomes in the same cluster. In this study, it is aimed at estimating to what extent the ANC, DPF, and C-section are inter-correlated in the cluster. To address this objective, a joint modelling approach is employed to model the ANC, DPF, and C-section variables simultaneously [24,25]. The developed joint model also facilitates measuring the joint association of predictors with the number of ANC, the likelihood of DPF, and the likelihood of C-section. The identified shared predictors of the ANC, DPF, and C-section from this study may provide an important understanding of the interplay among the considered maternal healthcare services.

## 2 Data and methodology

The data used in this study were extracted from the most recent Bangladesh MICS 2019, which is the sixth-round household survey in Bangladesh for collecting data on children and women's health and nutrition indicators at the national level. The MICS was carried out by the Bangladesh Bureau of Statistics (BBS) in collaboration with the United Nations International Children's Emergency Fund (UNICEF) Bangladesh as a part of the Global MICS Programme. Survey data were collected from 64 administrative districts in Bangladesh at the household level. The urban and rural areas within each district were identified as the main sampling strata, and the sample of households was selected in two stages. Within each stratum, a total of 3220 enumeration areas (EAs) or primary sampling units (PSUs) were selected systematically with a probability proportional to size. A systematic sample of 20 households was drawn from each of the sampled PSUs or EAs. Details of the survey design, sampling technique, questionnaire, and quality control are described in the Bangladesh MICS 2019 report [26].

The survey consisted of 53,716 ever-married women aged 15–49 years, who were eligible for asking maternal healthcare indicators. Nevertheless, only the women who had childbirth within the last two years of the survey date were considered for collecting pregnancy- and birth-related data. Finally, a total of 9285 women who provided information on ANC, DPF, and C-section for their last birth were included in this study. The women were asked how many times they visited medically trained personnel for ANC (content includes measuring blood pressure, taking urine samples, and taking blood samples), whether the delivery was conducted in PF (yes or no), and whether the delivery was done through C-section (yes or no). The women's responses on maternal healthcare services - ANC (count), DPF (categorical), and C-section (categorical) - were used as the outcomes for this study. Following the previous studies, several predictors such as region, place of residence, religion, household wealth status, husbands' education, and women's education, women's age at birth, and women's exposure to mass media associated with the outcomes were considered in this study [1,14,[16], [17], [18], [19]]. These considered variables are illustrated in a tabular format in Appendix Table 2 along with some previous studies where these variables were found to be associated with the three outcome variables of this study. The trends of the ANC, DPF, and C-section as well as their inter-correlations in Bangladesh during 2000–2019 were explored utilizing the five rounds of BDHS [8,11,[27], [28], [29]] and two rounds of MICS [30] datasets.

**Table 1:** Mean number of antenatal care (ANC) visits, proportion of delivery in private facilities (DPF), and caesarean section (C-section) by women's background characteristics in Bangladesh, N = 9285.

Characteristics	ANC (μ)	DPF (%)	C-section (%)	Sample
**Region**				
Barisal	2.68	28.02	27.78	835
Chattogram	2.79	29.23	25.86	1926
Dhaka	3.14	44.35	45.96	1795
Khulna	3.62	53.80	53.18	1275
Mymensingh	2.01	18.46	20.97	558
Rajshahi	2.84	39.96	40.59	956
Rangpur	3.06	30.22	31.54	1135
Sylhet	2.37	18.63	19.63	805
F**or**$\chi^{2}$**value (p-value)**	42.05 (<0.001)	496.19 (<0.001)	520.51 (<0.001)	
**Place of residence**				
Urban	3.85	43.80	46.39	1774
Rural	2.70	33.02	32.37	7511
F**or**$\chi^{2}$**value (p-value)**	368.27 (<0.001)	73.24 (<0.001)	124.03 (<0.001)	
**Religion**				
Muslim	2.92	34.70	34.63	8389
Others	2.88	38.62	38.95	896
**F-value/Chi-square (p-value)**	0.17 (0.677)	5.45 (0.020)	6.64 (<0.001)	
**Household wealth status**				
Poorest	1.81	13.49	13.40	2231
Poorer	2.36	25.87	26.67	1886
Middle	2.80	37.51	37.07	1810
Richer	3.55	46.38	46.44	1770
Richest	4.55	60.96	60.39	1588
F**or**$\chi^{2}$**value (p-value)**	459.04 (<0.001)	1097.71 (<0.001)	1069.56 (<0.001)	
**Women's education**				
No Education	1.55	10.76	12.17	846
Primary	2.15	19.34	19.39	2151
Secondary	3.06	38.14	37.43	4691
Higher	4.26	60.18	61.24	1597
F**or**$\chi^{2}$**value (p-value)**	408.63 (<0.001)	914.68 (<0.001)	919.22 (<0.001)	
***Mother's age at birth***				
Mean ± SE	20.08 ± 0.03	20.57 ± 0.06	20.72 ± 0.06	9285
**t-value (p-value)**	–	−10.11 (<0.001)	−13.10 (<0.001)	

**Husbands' education**				
No Education	2.28	24.82	23.83	2812
Primary	2.61	29.35	29.13	2794
Secondary	3.35	42.49	43.07	2603
Higher	4.31	58.81	60.39	1073
F**or**$\chi^{2}$**value (p-value)**	199.7 (<0.001)	499.6 (<0.001)	574.8 (<0.001)	
**Access to mass media at least once a week**				
No	2.19	21.42	21.30	3958
Yes	3.45	45.22	45.26	5327
F**or**$\chi^{2}$**value (p-value)**	719.3 (<0.001)	564.7 (<0.001)	572.7 (<0.001)	
**Total sample**	2.92	35.08	35.05	9285

**Table 2:** Variance-covariance of cluster-specific random effects, variance-covariance, and correlation $\left(\hat{\rho }\right)$ matrices for antenatal care (ANC), delivery in private facilities (DPF), and caesarean section (C-section) from the developed joint model.

	Variance-covariance of random effects			Variance-covariance of outcomes			Correlation of outcomes (p-value)		
	ANC	DPF	C-section	ANC	DPF	C-section	ANC	DPF	C-section
ANC	1.76			4.48			1.00		
DPF	1.78	1.81		2.44	22.88		0.24 (<0.001)	1.00	
C-section	0.23	0.23	0.11	2.50	19.30	22.32	0.25 (<0.001)	0.85 (<0.001)	1.00

### 2.1 Statistical methods

Since the MICS survey data were collected through a clustered sampling design, the ANC, DPF, and C-section outcome variables are clustered by nature. Hence, generalized linear mixed models (GLMMs) were developed, considering clusters have random effects on the outcome variables. At first, for each type of outcome variables, a separate GLMM was developed for identifying the corresponding significantly associated predictors. These separate GLMMs can answer which predictors are significantly associated with ANC, DPF, and C-section, ignoring the inter-correlation among the outcomes. However, our research interests are to assess the inter-correlation among the multiple outcome variables and to assess the influence of the predictors on the outcome variables simultaneously, which cannot be addressed by separate GLMMs [31,32]. Hence, a joint model is inevitable, and the joint model can be developed by combining the separate GLMMs after accounting for the inter-correlation among the outcome variables. Several approaches (shared parameters, random-effects models, etc.) are available in the literature to analyse the outcomes simultaneously, and each of the approaches has its own assumptions and limitations and can only be used when they completely fit with the research objectives. In this study, a cluster-specific random effect approach of the joint model has been applied assuming the outcomes within the cluster are correlated, with a less strict assumption on the association among the multiple outcomes [33].

To illustrate the implemented joint model, let assume $Y_{1ij}$, $Y_{2ij}$, and $Y_{3ij}$ denote the ANC, DPF, and C-section outcome variables for the $i^{th}$ women in the $j^{th}$ cluster respectively. Also let assume each outcome variable has a vector $X_{ij}$ of predictors. The joint model is then developed by joining the separate GLMMs as below:

$$\left(\begin{array}{c} \begin{array}{c} Y_{1ij}\\ Y_{2ij} \end{array}\\ Y_{3ij} \end{array}\right)=\left(\begin{array}{c} \begin{array}{c} \exp\left(\alpha_{0}+\alpha_{1}X_{ij}+b_{1i}\right)\\ \frac{\exp\left(\beta_{0}+\beta_{1}X_{ij}+b_{2i}\right)}{1+\exp\left(\beta_{0}+\beta_{1}X_{ij}+b_{2i}\right)} \end{array}\\ \frac{\exp\left(\gamma_{0}+\gamma_{1}X_{ij}+b_{3i}\right)}{1+\exp\left(\gamma_{0}+\gamma_{1}X_{ij}+b_{3i}\right)} \end{array}\right)+\left(\begin{array}{c} \begin{array}{c} \varepsilon_{1i}\\ \varepsilon_{2i} \end{array}\\ \varepsilon_{3i} \end{array}\right)$$

where $\alpha_{0},\beta_{0}\text{,}$ and $\gamma_{0}$ are the overall intercepts, $\alpha_{1},\beta_{1}\text{,}$ and $\gamma_{1}$ are the vector of regression coefficients, $b_{1i}$, $b_{2i}$, and $b_{3i}$ are the cluster-specific correlated random effects, and $\varepsilon_{1ij}$, $\varepsilon_{2ij}$, and $\varepsilon_{3ij}$ are individual specific uncorrelated random errors of the outcome variables. The above model indicates that $Y_{1ij}$ is assumed to follow a random intercept (cluster-specific) Poisson model and the other outcome variables are assumed to follow random intercept (cluster-specific) logistic models. The vectors of random effects $\left(b_{1i},b_{2i},b_{3i}\right)$ and random errors $\left(\varepsilon_{1ij},\varepsilon_{2ij},\varepsilon_{3ij}\right)$ are assumed to follow multivariate normal distribution with variance-covariance matrices denoted as below:

$$D=\left(\begin{array}{ccc} \delta_{1}^{2} & \delta_{12} & \delta_{13}\\ \delta_{12} & \delta_{2}^{2} & \delta_{23}\\ \delta_{13} & \delta_{23} & \delta_{3}^{2} \end{array}\right)\;\text{and}\;\Sigma_{i}=\left(\begin{array}{ccc} v_{1ij} & 0 & 0\\ 0 & v_{2ij} & 0\\ 0 & 0 & v_{3ij} \end{array}\right)$$

Following Fieuws & Verbeke [33] the approximate variance-covariance matrix of $Y_{1ij}$, $Y_{2ij}$, and $Y_{3ij}$ can be written as:

$${\begin{array}{c} V \end{array}}_{ij}=\Delta_{i}Z_{i}DZ_{i}^{\prime }\Delta_{i}^{\prime }+\Sigma_{i}=\left(\begin{array}{ccc} v_{1ij}^{2}\delta_{1}^{2}+v_{1ij} & \delta_{12}v_{1ij}v_{2ij} & \delta_{13}v_{1ij}v_{3ij}\\ \delta_{12}v_{1ij}v_{2ij} & v_{2ij}^{2}\delta_{2}^{2}+v_{2ij} & \delta_{12}v_{2ij}v_{3ij}\\ \delta_{13}v_{1ij}v_{3ij} & \delta_{12}v_{2ij}v_{3ij} & v_{3ij}^{2}\delta_{3}^{2}+v_{3ij} \end{array}\right)$$

where $Z_{i}=I_{33}$ and $\Delta_{i}=\Sigma_{i}$. Then the approximate correlation among the three outcomes can be calculated as:

$$\rho_{kl}=\frac{\delta_{\text{kl}}v_{kij}v_{lij}}{\sqrt{v_{kij}^{2}\delta_{k}^{2}+v_{kij}}\sqrt{v_{lij}^{2}\delta_{l}^{2}+v_{lij}}}$$

where k,l=1,2,3 and k≠l. In the case of $\rho_{kl}\equiv 0$, the above models in the joint model are equivalent to separate GLMMs for three outcome variables. The adaptive Gauss-Hermite quadrature in PROC GLIMMIX and adaptive Gaussian quadrature in PROC NLMIXED procedure of SAS 9.4 were utilized to estimate the parameters of the separate GLMMs and joint model respectively [22,34]. As the PROC NLMIXED is equipped with default starting values for integral approximation, user-defined starting values are required to ensure the stability of the iterative process [35]. At the beginning of this study, the separate GLMMs were fitted to use the output as starting values for the joint model and compare the coefficients with the estimates from the joint model.

## 3 Results

**Fig. 1:**
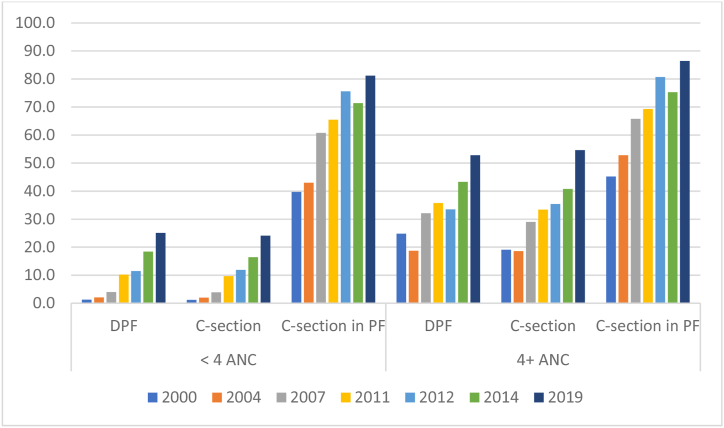
Trends of delivery in private facilities (DPF), caesarean section (C-section), and C-section in private facilities (PF) by women's antenatal care (ANC) during pregnancy for the period of 2000–2019 in Bangladesh. Data sources are 2000, 2004, 2007, 2011 and 2014 Bangladesh Demographic and Health Surveys; 2012 and 2019 Multiple Indicators Cluster Surveys.

Before developing the model, the maternal healthcare variables were thoroughly explored using descriptive statistics. Fig. 1 shows that the prevalence of DPF, C-section, and C-section in PF increased gradually over the period 2000–2019 for both groups of women who had lower (<4) and higher (4^+^) ANC visits. The prevalence of DPF and C-section was always substantially higher among the women with higher ANC visits compared to those with <4 ANC visits; however, the prevalence of C-section among the women who gave birth in PF was very similar for both groups. In 2019, more than 80 % of women who delivered birth in PF went through the C-section regardless of whether they had fewer ANC visits or more ANC visits. On the other hand, the prevalence of DPF and C-section (about 55 % for both indicators) was almost double for women who took 4^+^ ANC visits compared to women with <4 ANC visits (about 25 %).

Table 1 shows the mean number of ANC (counts), the prevalence of DPF (proportion), and of C-section (proportion) according to the background characteristics of women sampled in the MICS 2019 survey. To assess the statistical significance of the difference among several means and proportions, F and $\chi^{2}$ tests have been used, respectively. Women from the Khulna division had a higher mean ANC (3.62), a higher prevalence of DPF and C-section (53.80 % and 53.18 %, respectively), and those from the Mymensingh division had the lowest (2.01, 18.46 %, and 20.97 %, respectively). Notably, women in urban areas had higher ANC (3.85), DPF (43.80 %), and C-section (46.39 %) compared to rural women (2.70, 33.02 %, and 32.37 %, respectively). The mean ANC and prevalence of DPF and of C-section were higher for the women who had higher education (4.26, 60.18 %, and 61.24 %, respectively), more access to mass media (3.45, 45.22 %, and 45.26 %, respectively), and higher educated husbands (4.31, 58.81 %, and 60.39 %, respectively). Women from the richest families had a higher mean number of ANC visits (4.55) and higher proportions of DPF (60.96 %) and C-section (60.39 %). Muslim women had a slightly lower prevalence of DPF (34.70 %) and of C-section (34.63 %) compared to their counterparts (38.62 % and 38.95 %, respectively).

### 3.1 Model development

The separate GLMMs were developed using the same set of significant predictors, with all categories shown in Table 1 after accounting for the correlation among women in the same cluster. The associated results from the separate GLMMs are shown in Appendix Table 1. The estimated parameters from the separate GLMMs were used at first in the joint model to see whether the joint model can be fitted using PROC NLMIXED. Since the PROC NLMIXED did not support modelling with full parameterization, significant predictors were stratified in fewer groups according to their homogeneous distribution. For example, household wealth status was recategorized into two groups by considering the poorer and poorest as poor and others as rich or middle. Then the separate GLMMs were refitted with the new parameterization and combined into a joint model. The trial-and-error method was followed to adjust the estimates from the separate GLMMs, which were then utilized as the starting values in the joint model. This method was continued until the procedure was converged and the standard errors of all the estimates were available in the joint model.

### 3.2 Correlations between maternal healthcare indicators

**Fig. 2:**
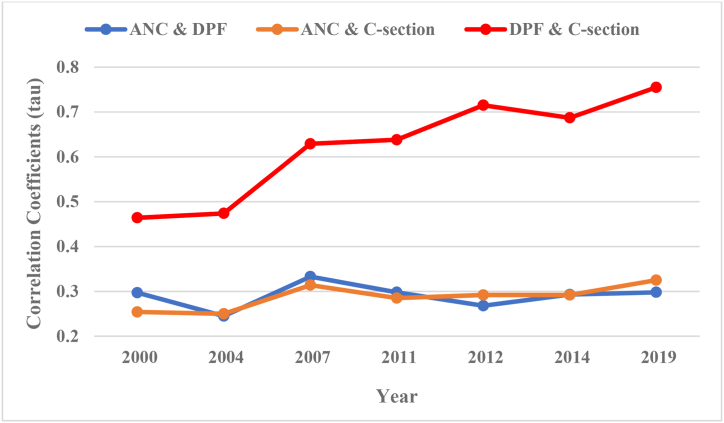
Trends of Kendall's tau correlation between the antenatal care (ANC), delivery in private facilities (DPF), and caesarean section (C-section) in Bangladesh. Data sources are 2000, 2004, 2007, 2011 and 2014 Bangladesh Demographic and Health Surveys; 2012 and 2019 Multiple Indicators Cluster Surveys.

Fig. 2 shows the correlations’ trend (calculated by Kendall tau statistics) for the pairs of ANC and DPF, ANC and C-section, and DPF and C-section over the period 2000–2019. Correlation of the pair DPF and C-section increased significantly (from about 0.45 in 2000 to about 0.75 in 2019) compared to the correlations of other pairs, which remained stable around 0.30 during 2010–2019.

The estimated variance-covariance matrix of the cluster-specific random effects from the developed joint model was utilized to approximate the variance-covariance and correlation matrices of the three outcomes ANC, DPF, and C-section, which are shown in Table 2. The significance of the estimated correlations indicated the necessity of considering a joint model rather than separate GLMMs. The model-based correlation of the pair DPF-C-section (ρ = 0.85, p-value = 0.001) was notably higher compared to the correlations of the pairs ANC-C-section (ρ = 0.25, p-value = 0.001) and ANC-DPF (ρ = 0.24, p-value = 0.001).

## 4 Significant predictors

Results of the developed separate GLMMs and the joint model are reported in Table 3. Incidence rate ratios (IRRs) obtained from the Poisson mixed model and odds ratios (ORs) obtained from the logistic mixed models are presented together with their corresponding Wald-type 95 % confidence intervals (CI) and p-values. The estimated IRRs for the number of ANC visits were very similar in separate GLMM and joint models; however, ORs of DPF and C-section obtained from the joint model were slightly different from those obtained from separate GLMMs. These differences may be because of the accounting correlation among the outcome variables in the model development. As the estimated correlations from the joint model were significant, the results of the joint model are considered benchmark results for this study, so we decided to explain them only. According to the joint model, division, place of residence, religion, household wealth status, women's age at birth, women and their partners' education status, and women's exposure to mass media have significant joint effects on the three outcome variables.

**Table 3:** Incidence rate ratio (IRR) of the number of antenatal care (ANC), odds ratios (ORs) of delivery in private facilities (DPF) and birth by caesarean section (C-section) based on the estimated regression coefficients of separate generalized linear mixed models (GLMMs) and joint model of ANC, DPF, and C-section.

Predictors	ANC				DPF				C-section			
	GLMM		Joint model		GLMM		Joint model		GLMM		Joint model	
	IRR (95 % CI)	p-value	IRR (95 % CI)	p-value	OR (95 % CI)	p-value	OR (95 % CI)	p-value	OR (95 % CI)	p-value	OR (95 % CI)	p-value
***Region (ref = Other divisions)***												
Dhaka	0.97 (0.93–1.01)	0.170	0.97 (0.93–1.02)	0.232	1.38 (1.20–1.60)	0.007	1.48 (1.23–1.78)	<0.001	1.51 (1.31–1.73)	0.001	1.66 (1.38–1.99)	<0.001
***Place of residence (ref =* Urban*)***												
Rural	0.83 (0.80–0.97)	<0.001	0.83 (0.79–0.86)	<0.001	1.03 (0.89–1.20)	0.901	0.97 (0.81–1.18)	0.785	0.88 (0.77–1.02)	0.092	0.80 (0.66–0.97)	0.020
***Religion (ref = others)***												
Muslim	1.02 (0.97–1.08)	0.340	1.02 (0.98–1.08)	0.363	0.78 (0.65–0.94)	0.004	0.77 (0.62–0.95)	0.014	0.80 (0.67–0.96)	0.018	0.76 (0.62–0.94)	0.011
***Household wealth status (ref =* Rich/Middle*)***												
Poor	0.76 (0.74–0.79)	<0.001	0.77 (0.74–0.79)	<0.001	0.43 (0.38–0.49)	<0.001	0.40 (0.35–0.46)	<0.001	0.48 (0.43–0.55)	<0.001	0.46 (0.40–0.53)	<0.001
***Women's education (ref = At least secondary)***												
At most primary	0.74 (0.72–0.77)	<0.001	0.75 (0.72–0.77)	<0.001	0.39 (0.34–0.44)	<0.001	0.37 (0.32–0.43)	<0.001	0.40 (0.36–0.46)	<0.001	0.39 (0.34–0.45)	<0.001
**Women's age at birth**												
	1.01 (1.01–1.02)	<0.001	1.01 (1.01–1.02)	<0.001	1.05 (1.03–1.07)	<0.001	1.06 (1.04–1.07)	<0.001	1.08 (1.06–1.09)	<0.001	1.09 (1.07–1.11)	<0.001
**Husbands' education *(ref = At least secondary)***												
At most primary	0.88 (0.85–0.90)	<0.001	0.88 (0.85–0.90)	<0.001	0.70 (0.63–0.78)	<0.001	0.70 (0.62–0.79)	<0.001	0.65 (0.58–0.72)	<0.001	0.64 (0.57–0.72)	<0.001
***Access to mass media at least once a week (ref = No)***												
Yes	1.21 (1.17–1.25)	<0.001	1.19 (1.16–1.23)	<0.001	1.81 (1.60–2.03)	<0.001	1.70 (1.49–1.94)	<0.001	1.80 (1.60–2.02)	<0.001	1.70 (1.49–1.94)	<0.001

The findings of the joint model indicate that the IRRs of ANC visits were significantly lower for the women who were from rural areas (0.83, CI: 0.79–0.86) and poor households (0.77, CI: 0.74–0.79), who had at most primary education (0.75, CI: 0.72–0.77), and who had partners with primary or less education (0.88, CI: 0.85–0.90). However, the IRR of ANC was significantly higher for the women who had access to mass media at least once per week (1.19, CI: 1.16–1.23) compared to their counterparts. The ORs of having DPF and birth by C-section were significantly higher for the women who lived in Dhaka division (DPF: 1.48, CI: 1.23–1.78 and C-section: 1.66, CI: 1.38–1.99) and who had access to mass media (DPF: 1.70, CI: 1.49–1.94 and C-section: 1.70, CI: 1.49–1.94), while ORs for DPF and C-section were significantly lower for the women from Muslim households (DPF: 0.77, CI: 0.62–0.95 and C-section: 0.76, CI: 0.62–0.94), the women living in poor households (DPF: 0.40, CI: 0.35–0.46 and C-section: 0.46, CI: 0.40–0.53), the women who had primary or no education (DPF: 0.37, CI: 0.32–0.43 and C-section: 0.39, CI: 0.34–0.45), and those women having less educated husbands (DPF: 0.70, CI: 0.62–0.79 and C-section: 0.64, CI: 0.57–0.72). Women's age at birth had a significant positive influence on the odds of having DPF (1.06, CI: 1.04–1.07) and a C-section (1.09, CI: 1.07–1.11). Rural women had non-significantly lower odds of having DPF (0.97, CI: 0.81–1.18) but had significantly lower odds of birth through C-section (0.80, CI: 0.66–0.97).

## 5 Discussion

The Multiple Indicator Cluster Survey (MICS) in Bangladesh uses a complex cluster sampling design to provide estimates on maternal healthcare indicators such as antenatal care, delivery in private facilities, and C-section at the national level. As these indicators are measured from the individuals in the same cluster, they are expected to inter-correlate with each other in the same cluster. However, all the past studies analysed the indicators of antenatal care, delivery in private facilities, and C-section utilizing the univariate approach, which ignores the potential correlation between the indicators in the same cluster [1,[14], [15], [16], [17], [18], [19],^36^,^37^]. Thus, the availability of clustered survey data on maternal healthcare services requires proper attention in regard to whether they are analysed using the appropriate statistical techniques. This study aimed to address this gap by modelling these indicators jointly, which ultimately facilitates to estimate their correlations, along with investigating their shared predictors in Bangladesh.

In the development process of separate GLMMs, the cluster-specific random effects were incorporated to prevent biased estimates with unfortunate under-coverage due to lower standard errors [38]. In the separate GLMMs, the count outcome (antenatal care) is assumed to follow a cluster-specific Poisson model, and the categorical outcomes (delivery in private facilities and birth through C-section) are assumed to follow cluster-specific logistic models. The separate GLMMs are then combined into a joint model by assuming a joint multivariate distribution on the cluster-specific random effects. Conditional upon the random effects, these three outcomes are assumed to be independent. The correlations between the outcomes are captured by letting cluster-specific random effects be associated [31]. The significance of the estimated pairwise correlations among the three outcome variables in the joint model indicates the necessity of using a joint model to identify their predictors simultaneously instead of a separate model for each of the outcome variables. The estimated strong positive correlation between delivery in private facilities and birth by C-section is an alarming issue to control the rate of C-section within the WHO recommended range of 10–15 %. It seems that mothers living in a particular cluster are more likely to use private facility care for their birth and may also seek C-section birth.

As the C-section is quite high in the private facilities, these facilities should be monitored with special attention to a large extent. Following the WHO recommended C-section rate and the guidelines for conducting C-sections (allowed only for justifiable complexity), hospital (both private and public) authorities can be assigned a maximum rate of C-sections per month based on their capacity and can be provided incentives for achieving the target. The hospitals that fail to meet the target or do not follow the restriction should be brought under strict accountability to the concerned authority. Most importantly, one-sided decisions on C-sections by practitioners (for whom doing C-sections is a profitable business) in private facilities should be discarded; in their place, shared decisions by practitioners and patients must be imposed strictly to stop unnecessary use of C-sections in private facilities.

The results obtained from the joint model show that women living in Dhaka division had significantly higher probabilities of giving birth in a private facility through C-section compared to those women living in other parts of the country. These findings are highly supported by the findings obtained in several previous studies [12,14,39]. The findings may suggest that the availability of private facilities in the capital city, the perception of providing improved care in private facilities, profit generative nature of private facilities towards the C-section delivery, and physician's influence on mother's choice to undergo for C-section birth are the contributing factors for the difference in maternal service utilization between the different geographic region [39]. On the other hand, the miserable condition of the public hospitals is the main reason for keeping away from them [40,41].

It is still observed that women living in urban settings are more likely to have a higher frequency of antenatal care visits [42] and hence are more likely to deliver birth in private facilities by C-section [12,18]. The results of this present study also show that women living in rural areas had about a 20 % lower incidence rate of taking antenatal care as well as a 20 % lower likelihood of giving birth by C-section. However, the odds of delivery in private facilities did not significantly vary by place of residence. The difference may arise due to the economic condition of rural married adolescents, more access to maternal health services among urban women, and the better education of urban women, who have comparatively better decision-making power on their own healthcare issues [43]. Religious and cultural beliefs of rural women may also be another reason for utilizing fewer maternal healthcare services [44].

It is still observed that women living in urban settings are more likely to have a higher frequency of antenatal care visits [42] and hence are more likely to deliver birth in private facilities by C-section [12,18]. The results of this present study also show that women living in rural area had about a 20 % lower incidence rate of taking antenatal care as well as a 20 % lower likelihood of giving birth by C-section. However, the odds of delivery in private facilities did not significantly vary by place of residence. The difference may arise due to the economic condition of rural married adolescents, more access to maternal health services among urban women, and the better education of urban women, who have comparatively better decision-making power on their own healthcare issues [43]. Religious and cultural beliefs of rural women may also be another reason for utilizing fewer maternal healthcare services [44].

This study also found that women belonging to Muslim families are about 25 % less likely to go to private hospitals for birth and to choose C-section birth compared to those from non-Muslim families. Some studies on maternal healthcare utilization support these findings [16,18,19,39]. These findings suggest that religious restrictions among Muslim women refrain them from going to health facilities, so they are at less risk of having birth by C-section [16,18].

The positive association of household wealth status with antenatal care, delivery in private facilities, and C-section has been evident in many studies [42,45]. This study also found that the incidence rate of taking antenatal care is about 25 % less and the probabilities of giving birth at a private facility and caesarean birth are about 60 % and 55 % less, respectively, among the women living in poor households compared to those of rich and middle-class households. A possible explanation could be that women belonging to well-off families usually have sufficient ability to spend more money for frequent antenatal care, to give birth in private hospitals with better facilities, as well as C-section birth.

Like other relevant studies [12,42,46], this study also found that higher-educated women are more likely to take an increased number of ANC services and to give birth in a private facility, as well as to give birth by C-section, compared to less-educated women. It is surprising that the risk of having a caesarean birth among women with at least secondary education is about 60 % higher than that of women with education up to the primary level. It is expected that educated women will take more ANC since they have sufficient knowledge of the benefits of ANC visits, such as reduction of pregnancy complications, endurance of a safe birth, and a healthier life for babies. However, they are also going through C-section, which highlights the knowledge gap on maternal care services, such as the short- and long-term impact of C-section on their health, their children, and future pregnancies [[47], [48], [49]]. Such results indicate that healthcare providers may not encourage pregnant women sufficiently for vaginal birth but instead may inspire C-sections by referring to this procedure as convenient, less painful, and relatively easier than vaginal birth [39]. In such a situation, educated women as well as women from well-off families are more likely to accept such advice from the healthcare provider [50].

Like women's education, partner's education has a very similar impact on taking ANC services, choosing private facilities as the place of birth, and choosing C-section as the birth method. However, the IRR and ORs are not as large as women's education. The wives of secondary or higher educated men were about 15 % more likely to have increased number of ANC visits, about 30 % more likely to choose private facilities for birth and about 25 % more likely to prefer C-section for the birth. Several previous studies reported a similar association [1,15,42]. Along with educated women, educated partners may also be concerned with their pregnant wives and the associated pregnancy complications.

The contribution of mass media to the maternal healthcare services was found to be like the contribution of women's education. The current study revealed that women who have access to mass media have a 20 % higher incidence rate of taking more ANC services and a 70 % higher chance of having both births in a private facility by C-section. These findings are also consistent with the relevant earlier studies [42,[50], [51], [52]]. Mass media broadcast different sorts of health-related programs and news that make women aware of their well-being and the well-being of their unborn baby.

The incidence rate of taking antenatal care, likelihood of birth in a health facility (including a private facility), and caesarean birth are positively associated with women's age at birth [16,18,39]. The current study also reported that the incidence rate of taking ANC, the odds of giving birth in a private facility, and the odds of having a caesarean birth increased linearly with the increase in women's age at birth. Women's higher education and involvement in income-generating activities may have an influence on this linear association of women's age at birth. It is also natural that women are more prone to complications at later stages of their lives [53], and so women who conceive babies at later stages of life are always worried about pregnancy complications and hence seek more antenatal care during the pregnancy, are more willing to use private health facilities, and opt for caesarean birth for the sake of safe birth and new-born health. Due to the unavailability of pregnancy complication information, their effects remained unexplained in the developed models but were captured by the model idiosyncratic error term. The modelling technique captures the simultaneous nature of correlations associated with each of the three outcome variables (ANC, DPF, and CS delivery). However, predominant predictors like socioeconomic status, education levels, and age of the women in the model explain the more frequent ANC visits significantly. Further, the large correlation between DPF and C-section delivery warns of a policy attempt to strengthen maternal care services. According to the World Health Organization, it is necessary to ensure that all women are able to talk to healthcare providers and be part of the decision-making process about their birth, receiving adequate information, including the risks and benefits. Emotional support is a critical aspect of quality care throughout pregnancy and childbirth. We conclude that there is an urgency for comprehensive research and policies on the determinants of increased CS delivery and policy attempts to bend the epidemic of CS in urban settings.

Even though this study shows controlling correlations do not change the effects of predictors much and draws conclusions from existing studies, it has some important contributions to existing studies. The main contribution of this study is to show how a joint model optimally uses the available information from multivariate maternal healthcare indicators occurring simultaneously rather than utilizing them in univariate form. Another contribution of this study to the literature is to show a scientific way to assess the correlation among three maternal care indicators after adjusting the influence of the explanatory variables. This study utilizes the most recent large survey data, which is representative at the second administrative level (district). So, the findings of this study based on Bangladesh data are up-to-date and expected to reveal the scenario of south and south-east Asian countries where the C-section rates are higher than the WHO recommended optimal range [13].

The study also has some limitations, which include justification of the distribution of the random effects, less feasibility of considering complex models with many parameters, re-parametrization of the covariates (which can misrepresent the real scenario), and the time-consuming nature of the PROC NLMIXED procedure. However, it can be expected that the estimated regression coefficients of the joint model are approximately unbiased if distributions of random effects are incorrectly specified [54]. Some potential associated factors, such as women's working status and complications during pregnancy and at birth, were not utilized in this study as they were not available in the dataset, which might have implications for the conclusion and policy recommendation. This provides an opportunity for future research that can focus on overcoming these limitations (for instance, including more potential covariates in the joint model, finding a way to add covariates with full parametrization, etc.) of this study.

## 6 Conclusions

Based on the study findings, several concluding remarks and recommendations can be drawn for policymakers and stakeholders who work in the health sector. The very similar positive correlation of antenatal care with delivery in private facilities and C-section may be reasonable, but it is necessary to examine whether getting access to more antenatal care has any indirect influence on the high association between delivery in private facilities and C-section. In this regard, policy makers may focus on the antenatal care services to adapt the contents by including proper consultancy by the healthcare providers (doctors, nurses, midwives, and others) to motivate women (particularly those who have decided to give birth in private facilities) to do vaginal delivery instead of C-section unless they have complications. Moreover, policymakers should promote and improvise community-based education programs to raise awareness about the importance of vaginal deliveries and C-sections, focusing on early and regular antenatal check-ups to reduce unnecessary C-sections. Since the C-section rate is increasing day by day in Bangladesh, the government can take the necessary steps to issue uniform evidence-based clinical guidelines to both government and private hospitals or clinics to reduce the number of unnecessary C-sections.

The study findings also suggest focusing on the subgroups (for instance, women who have sufficient education, higher educated partners, and sufficient economic shelter) who are likely to take more antenatal care, give birth in private facilities, and opt for C-section as the best and safest mode of birth. It is essential to take the necessary steps to educate the pregnant women and their family members about the benefits (short-run) and adverse consequences (long-run) of the C-section on their lives and the lives of new-borns, along with the economic burden.

## Data availability statement

The detailed microdata of Bangladesh Multiple Indicators Cluster Surveys and Bangladesh Demographic and Health Surveys can be accessed through https://mics.unicef.org/and https://dhsprogram.com/respectively after proper registration. Since microdata of both types of surveys are accessible but the authors do not explicit permission to share the detailed microdata publicly, only the final dataset consisting of the considered variables used in developing the models has been submitted to this journal as an additional supporting file with the manuscript. For reproducibility purpose, the core SAS codes for fitting the models are also supplied in an additional supporting file with the manuscript.

## Ethical issues

The datasets used in the present study do not contain any individual identifier and are freely available on the MICS and the DHS websites. Hence, this study does not require research and ethical review approval. However, verbal consent was obtained from each participating woman, and the confidentiality and anonymity of information were informed to them at the time of survey.

## Funding

This research did not receive any grant.

## Questionnaire

Full questionnaire of the household surveys are available in MICS and DHS reports.

## CRediT authorship contribution statement

**Kakoli Rani Bhowmik:** Writing – review & editing, Writing – original draft, Methodology, Formal analysis, Data curation, Conceptualization. **Sumonkanti Das:** Writing – review & editing, Writing – original draft, Visualization, Validation, Supervision, Software, Methodology, Conceptualization. **Unnati Rani Saha:** Writing – review & editing, Validation, Supervision, Methodology, Investigation, Conceptualization. **Ruhul Amin:** Writing – review & editing, Visualization, Software, Resources, Methodology, Data curation. **Md Atiqul Islam:** Writing – review & editing, Visualization, Validation, Supervision, Software, Methodology, Conceptualization.

## Declaration of competing interest

The authors declare that they have no known competing financial interests or personal relationships that could have appeared to influence the work reported in this paper.

## Figures and Tables

**Appendix Table 1:** Incidence rate ratio (IRR) of the number of antenatal care (ANC), odds ratios (ORs) of delivery in private facilities (DPF) and birth by caesarean section (C-section) based on the estimated regression coefficients of separate generalized linear mixed models (GLMMs) of ANC, DPF, and C-section developed using the detailed categories of predictors

	ANC		DPF		C-section	
	IRR (95 % CI)	p-value	OR (95 % CI)	p-value	OR (95 % CI)	p-value
Division (ref = Barisal)						
Chattogram	0.88 (0.82–0.95)	<0.001	0.68 (0.54–0.86)	0.002	0.59 (0.47–0.74)	<0.001
Dhaka	0.92 (0.86–0.98)	0.015	1.27 (1.01–1.61)	0.039	1.42 (1.13–1.78)	0.002
Khulna	1.15 (1.07–1.23)	<0.001	2.31 (1.81–2.93)	<0.001	2.31 (1.83–2.91)	<0.001
Mymensingh	0.76 (0.69–0.84)	0.156	0.55 (0.39–0.76)	<0.001	0.68 (0.49–0.92)	0.012
Rajshahi	0.95 (0.88–1.02)	0.006	1.41 (1.08–1.81)	0.009	1.49 (1.17–1.91)	0.001
Rangpur	1.11 (1.03–1.19)	<0.001	0.99 (0.77–1.27)	0.931	1.10 (0.86–1.40)	0.445
Sylhet	0.85 (0.78–0.92)	<0.001	0.39 (0.29–0.54)	<0.001	0.45 (0.34–0.61)	<0.001
**Place of residence (ref = Urban)**						
Rural	0.90 (0.86–0.94)	<0.001	1.33 (1.14–1.55)	<0.001	1.07 (0.92–1.24)	0.362
**Religion (ref = Non-Muslim)**						
Muslim	0.99 (0.94–1.04)	0.775	0.68 (0.56–0.82)	<0.001	0.69 (0.58–0.83)	<0.001
**Household wealth status (ref = Richest)**						
Poorest	0.57 (0.53–0.60)	<0.001	0.16 (0.12–0.20)	<0.001	0.19 (0.15–0.24)	<0.001
Poorer	0.64 (0.61–0.68)	<0.001	0.26 (0.21–0.32)	<0.001	0.32 (0.26–0.39)	<0.001
Middle	0.72 (0.69–0.75)	<0.001	0.39 (0.33–0.47)	<0.001	0.45 (0.38–0.55)	<0.001
Richer	0.84 (0.81–0.88)	<0.001	0.52 (0.43–0.61)	<0.001	0.59 (0.50–0.70)	<0.001
**Mother's education (Ref = Higher education)**						
No Education	0.60 (0.56–0.64)	<0.001	0.22 (0.16–0.30)	<0.001	0.26 (0.20–0.34)	<0.001
Primary	0.75 (0.71–0.78)	<0.001	0.36 (0.30–0.44)	<0.001	0.37 (0.30–0.45)	<0.001
Secondary	0.89 (0.86–0.92)	<0.001	0.64 (0.56–0.75)	<0.001	0.64 (0.55–0.74)	<0.001
**Women's age at birth** (Continuous variable)						
	1.009 (1.005–1.013)	<0.001	1.04 (1.03–1.06)	<0.001	1.07 (1.05–1.09)	<0.001
**Husbands' education (Ref = Higher Education)**						
No Education	0.91 (0.86–0.95)	<0.001	0.82 (0.68–0.99)	0.049	0.71 (0.59–0.90)	0.001
Primary	0.93 (0.89–0.98)	0.003	0.82 (0.67–0.98)	0.035	0.75 (0.62–0.90)	0.003
Secondary	0.99 (0.95–1.03)	0.584	0.95 (0.80–1.14)	0.629	0.93 (0.79–1.11)	0.475
***Access to mass media at least once a week (ref = No)***						
Yes	1.14 (1.10–1.18)	<0.001	1.42 (1.26–1.61)	<0.001	1.41 (1.25–1.58)	<0.001

**Appendix Table 2:** Considered predictor variables and their association with the outcome variables (ANC = Antenatal care, DPF = delivery in private facilities and CS= Caesarean section) of maternal health care services along with references, data sources, and study findings.

Variable	Description	Author [reference number] and Data Sources	Outcome Variable	Relationship
**Region**	Administrative level 1 known as Division. There are 8 divisions as per MICS 2019: Barisal, Chattogram, Dhaka, Khulna, Mymensingh, Rajshahi, Rangpur, & Sylhet	Bhowmik et al. (2020) [42] & 2014 BDHS	ANC	Women belong to the Sylhet and Chittagong regions are less likely to use ANC and also have fewer ANC visits. While Khulna and Rangpur has higher incidence rate of taking more ANC visits.
		Huda et al. (2019) [16] & 2010 BMMS	DPF	The odds of using DPF for child birth was found significantly higher for Dhaka, Khulna and Rajshahi compared to Sylhet and Chittagong.
		Ahmed et al. (2022) [14] & 2019 MICS; Kamal (2013) [18] & 2007 BDHS	CS	The odds of having C-Section is found significantly low in Chittagong and Sylhet compared to other divisions.
		Kamal (2013) [18] & 2007 BDHS	CS	Higher odds of opting CS is found women from Dhaka and Khulna
Place of residence	It indicates cluster level residential information - Urban and Rural.	Bhowmik et al. (2020) [42] & BDHS, 2014	ANC	Rural women had lower incidence rate of taking higher number of ANC visits and higher odds of having no ANC visits.
		Chanda et al. (2020) [55] & 2014 BDHS	ANC	Women from urban areas had higher possibility to have 8 or more ANC contacts than their rural counterparts.
		Huda et al. (2019) [16] & 2010 BMMS	DPF	The odds ratio of using DPF is significantly higher among urban women than rural women
		Rahman et al. (2018) [1] & 2014 BDHS	CS	Urban women had higher possibility to opt CS for child birth
Religion	Since more than 90 % population are Muslim, two levels are assumed as Muslim and non-Muslim.	Chanda et al. (2020) [55] & 2014 BDHS	ANC	Muslim women had lower possibility to contact at least 8 ANC contacts
		Setu et al. (2022) [15] & 2017-18 BDHS; Huda et al. (2019) [16] & 2010 BMMS	DPF	Non-Muslim women had significantly higher odds of taking DPF for child birth compared to Muslim women.
		Kamal 2013 [18] & 2007 BDHS	CS	Non-Muslim women had significantly higher odds of taking CS for child birth compared to Muslim women.
Household wealth status	The households are divided in five groups (Poorest, poorer, middle, richer, and richest) based on their wealth index.	Bhowmik et al. (2020) [42] & BDHS, 2014	ANC	The odds ratios of having no ANC during pregnancy significantly reduces with the household wealth status, while women in rich households had greater possibility to take higher ANCs.
		Chanda et al. (2020) [55] & 2014 BDHS	ANC	Odds of ANC contacts increase with the household wealth status
		Setu et al. (2022) [15] & 2017-18 BDHS; Huda et al. (2019) [16] & 2010 BMMS	DPF	Odds ratio of using DPF increases with the household wealth status.
		Ahmed et al. (2022) [14] & 2019 MICS; Rahman et al. (2018) [1] & 2014 BDHS; Kamal 2013 [18] & 2007 BDHS	CS	Women from rich households had higher possibility to opt CS for child birth
Women's education	Four categories are: Illiterate, primary, secondary, and higher education	Bhowmik et al. (2020) [42] & BDHS, 2014	ANC	Incidence rate ratio of taking higher number of ANC visits and odds ratio of not taking any ANC respectively increases and decreases with the women education status.
		Setu et al. (2022) [15] & 2017-18 BDHS; Huda et al. (2019) [16] & 2010 BMMS	DPF	Women with higher education had at least 50 % higher possibility (OR>1.5) for child birth in DPF than their counterparts.
		Rahman et al. (2018) [1] & 2014 BDHS;	CS	Maternal education found positively associated with CS but found statistical non-significant after controlling other variables.
		Kamal 2013 [18] & 2007 BDHS	CS	Maternal education found positively associated with CS after controlling other variables
Husbands' education	Four categories are: Illiterate, primary, secondary, and higher education	Bhowmik et al. (2020) [42] & BDHS, 2014	ANC	Odds ratio of not taking any ANC visits is significantly lower for the husbands with secondary and higher education.
		Chanda et al. (2020) [55] & 2014 BDHS	ANC	Women with at least secondary educated husband had higher possibility to have ANC contacts
		Setu et al. (2022) [15] & 2017-18 BDHS	DPF	Husbands with higher education had odds ratio of 1.4 for child birth in DPF than their counterparts.
		Rahman et al. (2018) [1] & 2014 BDHS	CS	Husbands' education status found positively associated with CS but found statistical non-significant after controlling other variables.
Women's age at birth	Based on women's birth history.	Chanda et al. (2020) [55] & 2014 BDHS	ANC	Women aged 20–34 had significantly higher possibility to have ANC contact.
		Setu et al. (2022) [15] & 2017-18 BDHS	DPF	Women age more than 18 years at their first child birth had odds ratio of 1.4 for child birth in DPF than their counterparts.
		Ahmed et al. (2022) [14] & 2019 MICS; Rahman et al. (2018) [1] & 2014 BDHS	CS	Women aged 30 years and older had more possibility to use CS for child birth.
		Kamal 2013 [18] & 2007 BDHS	CS	Women aged 19–34 had higher possibility to have CS
women's exposure to mass media	Access to mass media (radio, television, and other media)	Bhowmik et al. (2020) [42] & BDHS, 2014; Chanda et al. (2020) [55] & BDHS, 2014	ANC	Women with exposure to media at least once a week had significantly greater possibility to attend more ANC visits.
		Setu et al. (2022) [15] & 2017-18 BDHS; Huda et al. (2019) [16] & 2010 BMMS	DPF	Women access to media had about 25 % higher possibility to use DPF for their child birth.
		Ahmed et al. (2022) [14] & 2019 MICS	CS	Women watch television almost every day had greater odds to opt CS

BDHS: Bangladesh Demographic and Health Survey; BMMS: Bangladesh Maternal Mortality Survey; MICS: Multiple Indicator Cluster Survey.
